# EMV-Compatible Offline Mobile Payment Protocol with Mutual Authentication

**DOI:** 10.3390/s19214611

**Published:** 2019-10-23

**Authors:** Jia-Ning Luo, Ming-Hour Yang

**Affiliations:** 1 Department of Information and Telecommunications Engineering, Ming-Chuan University, Taoyuan 33350, Taiwan; 2 Department of Information and Computer Engineering, Chung Yuan Christian University, Taoyuan 32023, Taiwan

**Keywords:** NFC, EMV, mobile payment, reverse hash chain

## Abstract

In 2014, Yang proposed a method to enhance the current EMV credit card protocol (EPMAR). However, the protocol ignores the exceeding of a credit quota caused by multiple offline transactions, with the result that the amount spent can exceed the risk control scope. In this paper, we proposed an EMV-compatible offline mobile payment protocol with mutual authentication (EOPMA) to enhance EPMAR. In EOPMA, we use the reverse hash chain technique to guarantee the payment, which solves the problem of credit quotas getting exceeded because of multiple offline payments. During a transaction, in addition to payment for merchandise, an offline authorization certificate for the transaction is sent to the merchant. The merchant can verify the correctness of the transaction in real time. Our protocol is compatible with the EMV standard, which is applicable to the retail environment of numerous merchants and effectively, making EMV transactions more secure and reliable. We use numerical analysis to examine the security and performance of the protocols. We formally check the correctness of EOPMA by using the Gong–Needham–Yahalom logic.

## 1. Introduction

Credit cards have become crucial transaction tools. In 2002, the standards for EMV chip credit cards were set by international organizations such as Europay, MasterCard, and Visa [1,2]. Chip credit cards contain a microprocessor for computing power and a tamper-proof space for storing encryption keys and personal information. Scholars have investigated potential methods of improving the security of EMV protocol. For example, Ruiter and Poll applied the EMV protocol to their standardized modules [3] and used a third-party verification tool to formally analyze and validate the EMV protocol. Chen et al. proposed an improvement to the EMV key generation mechanism [4]. Murdoch and Anderson mentioned that the EMV protocol may come under threat and thus proposed improvements for mitigating threats to the EMV protocol [5]. Moreover, Alhothaily proposed a user-controlled multiple-condition verification method for improving security [6], solving the problem of a simple card verification method.

Contactless chip credit cards that employ near-field communication (NFC) sensing technology have gradually become mainstream [7,8,9,10,11]. MasterCard and Visa have created contactless credit cards, namely PayPass [12] and payWave [13], respectively. Because of the increasing popularity of NFC smartphones, Steffens [14], Cheng [15], and Noh et al. [16] proposed the integration of credit cards into mobile phones. Google, Microsoft, and Apple Inc. have also implemented a mobile phone virtual credit card mechanism [17,18,19,20] to replace conventional chip credit cards. Users only need an NFC smartphone with a virtual credit card to make a purchase, not needing to carry a physical chip card.

Numerous scholars have suggested security enhancements, analyzed smartphone NFC-based credit cards [21,22,23,24,25,26,27,28,29], and attempted to implement EMV credit cards on NFC smartphones to achieve convenience and security. Pasquet et al. proposed a security framework for detecting security issues with NFC smartphone credit cards [20] (e.g., transactions may be blocked or forged, privacy protection of secure element’s SIM card owner, protection of essential transaction data, transaction application security, hardware tamper-proof protection mechanism, and protection of personal data), and verified the detection processes using detection tools. Furthermore, Paillès et al. [22] proposed that verification messages be separately sent to both merchant and issuer, with the merchant not told the identity of consumer, but the consumer’s identity is verified by the issuer. Mainetti et al. suggested a peer-to-peer message exchange method when exchanging messages between NFC smartphones and merchants’ points of sale (POSs) [23]. Moreover, Urien and Piramuthu proposed that the secure element in an NFC phone be replaced with a cloud-based security element that provides security services to properly implement the EMV credit card protocol [24].

In addition, scholars have stated that NFC faces the following security threats [30,31,32,33]: (1) NFC is a wireless transfer method in which an electromagnetic wave is received by NFC devices nearby when a message is sent; malicious users can thus eavesdrop and obtain the message. (2) A malicious user can attempt to modify the message content. (3) A malicious user can disturb the NFC-transmitted message and corrupt it, resulting in an inability of the NFC card reader to interpret the message and thus denial of service. Finally, (4) a malicious user can determine the location of a particular NFC device because the identification number of each NFC device is unique. When an EMV transaction is conducted offline (e.g., when on an airplane), the merchant is unable to confirm the validity of a virtual credit card with the issuer in a timely manner as is the case in an online transaction; malicious users can thus commit fraud [34]. The offline risk control mechanism described in the EMV protocol cannot prevent a malicious user from committing fraud if the transaction amount is below a threshold [35]. Some studies have attempted to increase the security of offline transactions; for instance, Blaze et al. proposed risk control mechanisms such as adding a limit to the consumption amount and usage time to users’ certificates obtained from the issuer to reduce the offline payment risk [36]. Rivest and Shamir suggested that users first apply for a certificate that has an expiration date and credit quota from the issuer before making any transactions. A PayWord that does not exceed the credit quota is generated when an offline purchase is made [37]. In research on PayWord [37,38,39,40,41], approaches have mainly been suggested that can only be used for single-merchant restrictions, but some have also been proposed for multiple merchant restrictions [42,43,44].

Yang proposed EMV-based Payment with Mutual Authentication and Risk management (EPMAR), which is suitable for both offline and online transactions [45]. EPMAR adds mutual authentication to the original EMV protocol in a compatible manner to solve the problem that anyone can use a POS to read a card [46,47]; in EPMAR, all messages are encrypted using a shared key so that a malicious user cannot determine the content even if they hack into the transaction message [48,49]. However, the disadvantage of EPMAR is that the transaction amount exceeds the risk control range allowed by the credit card after multiple offline transactions.

In this paper, we propose EMV-based Offline Payment with Mutual Authentication (EOPMA), which is based on EPMAR and compatible with EMV mutual authentication in the NFC smartphone environment. In EOPMA, an offline transaction certificate and reverse hash chain are used to split and control transaction amounts for offline transactions, and the hash value obtained in each transaction clarifies the amount already spent. A counter installed in the NFC mobile device’s secure element is employed in the credit control method. The counter is forced to increase according to the amount spent in each transaction. Before an offline transaction is completed, the user must apply for an offline certificate that stipulates their credit quota and transaction authorization from the issuer; merchants can thus verify correctness of an EMV offline transaction.

Through a issuer’s endorsement value and the verification message given to each participating party (merchant and issuer), and the issuer can check the content of all transactions and verify the correctness of a message’s content (including the verification message sent by the user to the merchant), making transactions more secure by employing layers of checks.

EOPMA solves the exceeding of a credit quota caused by duplicate transactions in EPMAR that is beyond risk control to enhance the security of offline transactions. For the remainder of this paper, Section 2 introduces the EOPMA proposed in this study; Section 3 proves the security analysis of the protocol, and compares the performance with that proposed in other studies; Section 4 summarizes the methods proposed and the contributions.

## 2. EMV-Based Offline Payment with Mutual Authentication

**Figure 1:**
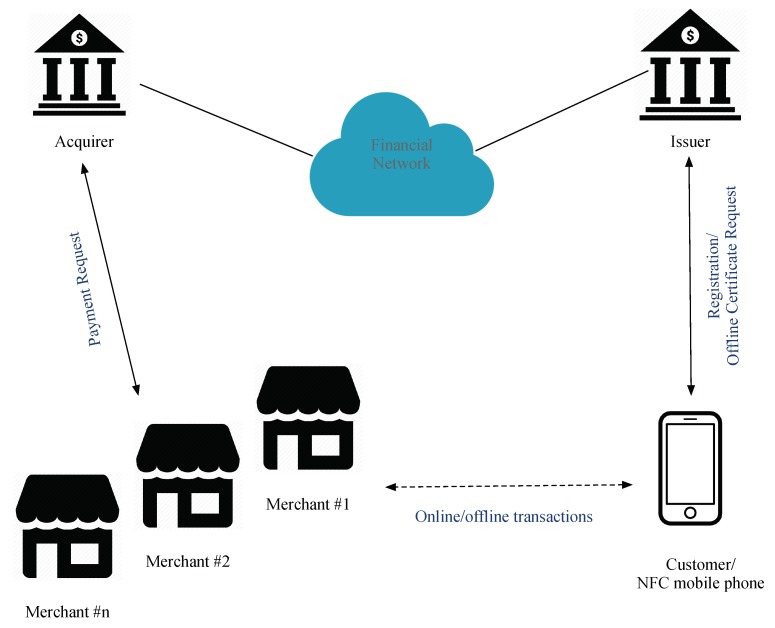
EOPMA infrastructure.

An offline mutual authentication mobile payment protocol compatible with EMV is proposed in this study and is called EOPMA. EPMAR, the basis for EOPMA [45], increases the security of offline transactions and is applicable to NFC smartphones. The parties involved in EOPMA are the issuer, NFC mobile phone, acquirer, and merchant, as shown in Figure 1. Their roles are as follows:

1. Issuer: responsible for managing the application of credit cards and the issuance of offline transaction certificates. The issuer also communicates with the acquirer that deploys POS terminals through a secured financial network;
2. NFC mobile phone: used to store a virtual credit card in a secure element, and inductively transfers transaction data with the merchant’s POS. When a user wants to make an offline transaction, he must first apply for an offline transaction certificate from the issuer.
3. Merchant: deploys a POS to read the NFC mobile phone; transaction data received by the merchant are transmitted to the acquirer through a secure channel. Merchants can conduct online or offline transactions with the user.
4. Acquirer: receives the transaction data from the merchant and confirms the correctness of the transaction through the financial network. In offline transactions, the merchant cannot connect the acquirer to check if a credit card has been revoked.

**Figure 2:**
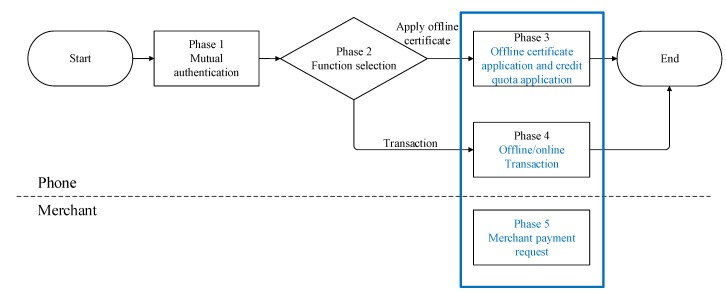
EOPMA flowchart.

The EOPMA process is divided into five phases: mutual authentication of the mobile phone, function selection, offline certificate application and split credit quota authorization, offline and online transactions, and payment request by merchant, as shown in Figure 2. The phases are explained as follows:

In phase 1 *Mutual authentication*, mutual authentication of the EMV card and POS is performed, and the exchanged certificates of both user and merchant are authenticated. The EMV card and POS verifies each other’s identity. Otherwise, the EMV card returns a failed acknowledgement and aborts the transaction.

Phase 2 is the function selection phase in which the merchant checks whether the user already has an offline certificate. In phase 2, two functions can be selected: applying offline certificate (go to phase 3), or go transactions (phase 4). Phases 1 and 2 are similar to those in EPMAR and the details are thus not discussed in this paper.

In phase 3, the user requests an offline transaction certificate and the amount required for the transaction from the issuer. In addition to an offline certificate, a credit quota calculated by the issuer is obtained from the application to effectively control the offline transaction amount. Moreover, crucial messages are protected by a secure element.

In phase 4, the transaction process begins. The EMV chip specifications define two types of the card authentication: online transaction and offline transaction. The processing steps for an EMV contact chip transaction are defined in Figure 4 of [2]. If the NFC mobile phone requests to go online, then the merchant’s terminal builds an online request to the issuer via the financial bank network for online card authentication. Otherwise, an offline transaction is performed if the user has a certificate; in an offline transaction, the merchant verifies the transaction message transferred by the user; if the merchant supports online transactions, the transaction message received by the merchant is transferred to the acquirer for verification. In phase 4, the mobile phone uses the credit quota parameters obtained during the application to calculate the amount spent in the current transaction and to generate a unique verification message for sending to the merchant and issuer. The merchant immediately knows the correct transaction amount, whereas the issuer is informed of the correct credit quota and the identity of the transaction merchant.

Phase 5 is the phase in which the merchant requests payment from the issuer. In phase 5, after the offline transaction has been completed, the merchant requests payment from the issuer via the acquirer by using the transaction verification data provided by the user, and the issuer uses the verification message sent by the user to the merchant via the acquirer to confirm whether the transaction is legitimate.

### 2.1. Generation of Offline Transaction Certificates

The method proposed in this study is compatible with the EMV standards [1]. The amount available for offline transaction is represented as two usable hash values that clearly indicate the amount used and amount usage range. Both merchants and issuers can verify the availability of this credit quota. The user must first apply for an offline transaction certificate with the issuer before making an offline transaction. During the transaction, the user shows the certificate to the merchant so that the merchant can submit the certificate to the issuer via the acquirer to verify the validity of the offline transaction.

#### 2.1.1. (a) Generation of Secret Factors

The secret factors $w_{0}$ and $s_{n}$ used in the credit hash chain are not randomly generated but obtained using the method employed by PayFair [25] and proposed by Yen et al. Before the issuer generates $w_{0}$ and $s_{n}$, it first generates the random numbers $R_{w}$ and $R_{s}$ and corresponding sequence numbers $SN_{w}$ and $SN_{s}$. It then stores $\left\{SN_{w},R_{w}\right\}$ and $\left\{SN_{s},R_{s}\right\}$ in a table, as illustrated in Table 1.

The issuer uses the uniquely owned key $K_{iss}$ to encrypt $\left\{SN_{w},R_{w}\right\}$ and $\left\{SN_{s},R_{s}\right\}$ into the $w_{n}$ and $s_{0}$ of the hash chain to form $w_{n}=E_{K_{iss}}\left(SN_{w},R_{w}\right)$ and $s_{0}=E_{K_{iss}}\left(SN_{s},R_{s}\right)$. During issuer verification of the merchant’s payment request phase, the issuer receives $SN_{w}$ and $SN_{s}$ to identify $R_{w}$ and $R_{s}$ and uses $K_{iss}$ to calculate the $w_{n}=E_{K_{iss}}\left(SN_{w},R_{w}\right)$ and $s_{0}=E_{K_{iss}}\left(SN_{s},R_{s}\right)$ to obtain the correct $w_{n}$ and $s_{0}$ for starting the verification process.

#### 2.1.2. (b) Credit Quota Calculation Method

Assuming the available credit limitation is *n*, the issuer calculates the entire hash chain $w_{0},w_{1},\cdots ,w_{n}$ by using the calculated $w_{n}$:

(1) $$w_{i}=h\left(w_{i+1}\right),\forall i=n-1,n-2,\cdots ,0.$$

#### 2.1.3. (c) Issuance of Offline Certificates and Credit Quota Calculation Method

When applying for an offline transaction, the issuer gives the user an offline certificate $Cert_{off}$ and a credit quota Lim. In the offline certificate, the user is informed of their upper credit limit *n*, and the amount of the secret value $w_{n}$, $w_{0}$ of the endorsed $\varphi =E_{SK_{iss}}\left(w_{0}\right)$, and $SN_{w}$ for verification are placed in the credit quota content. The user employs the same calculation method as the issuer, $w_{i}=h(w_{i+1}$, for i=n−1,n−2,⋯,0 and uses $w_{n}$ to calculate the entire series of hash chains $w_{0},w_{1},\cdots ,w_{n}$. The user verifies $w_{n}$ through *n* depending on whether the calculation result of the hash function, $w_{0}$, is the same to the $w_{0}$ endorsed by the issuer.

#### 2.1.4. (d) Use of the Credit Quota

In each transaction, the amount spent is split from the credit quota to pay the merchant. The splitting method is to sequentially give the hash value from the hash chain from $w_{0},\cdots ,w_{b},$$\cdots ,w_{b+c},\cdots ,w_{n}$; when it reaches $w_{n}$, the maximum credit quota has been reached. To make further purchases, the user must reapply a new quota to the issuer.

**Figure 3:**
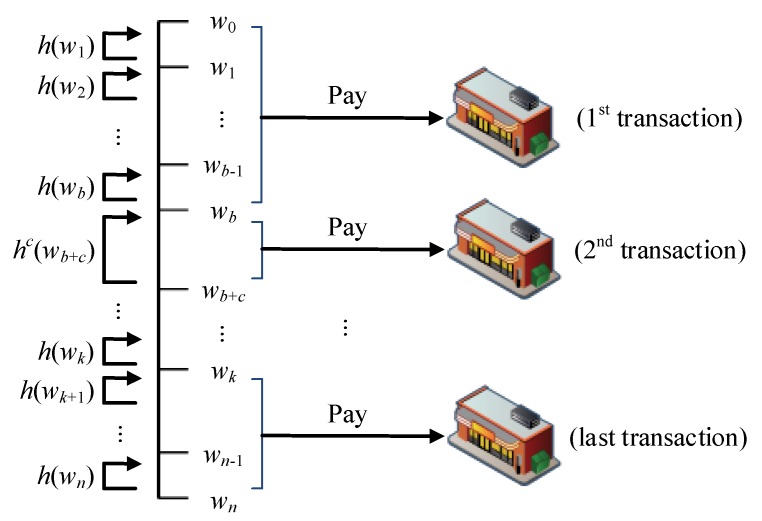
Simple offline transaction credit splitting diagram.

The hash value generated by $w_{i}=h\left(w_{i+1}\right)$ is used to represent the currently spent amount, and the interval between $w_{b}$ and $w_{b+c}$ is used to perform *c* calculations to obtain the amount used in the transaction. As illustrated in Figure 3, assuming that the offline credit quota applied is *n* and the amount spent in the first transaction is *b*, the user provides $w_{0}$ and $w_{b}$. In the second transaction, the amount spent is *c*, and the user provides a continued amount of use of $w_{b}$ and $w_{b+c}$. Finally, as shown in Figure 3, the credit quota is reached after multiple transactions when the user gives the merchant $w_{k}$ and $w_{n}$, at which point the user must reapply for an offline transaction certificate to the issuer.

This protocol employs the characteristics of the reverse hash chain, with which the merchant obtains $w_{i}=h\left(w_{i+1}\right)$; the merchant cannot calculate the previous hash value $w_{i+1}$ and thus cannot forge any amount unequal to that the user has authorized to the merchant. The protocol applies to a retail environment in which many merchants may be involved. When the user makes their first purchase, the merchant is given a quota between $w_{b}$ and $w_{b}$, whereas, when the second purchase is made, the second merchant will be given the limit of $w_{b}$ and $w_{b+c}$. This method is used to achieve an offline transaction mechanism that can be used for multiple merchants.

#### 2.1.5. (e) Credit Quota Verification

As illustrated in Figure 3, the amount spent in the first transaction is *b*, and the merchant obtains $w_{0}$ and $w_{b}$ from the user. The merchant can judge the correctness of $w_{b}$ by calculating whether $w_{b}$ after *b* hash function calculations is equal to $w_{0}$. The amount spent in the second purchase is *c*, and the merchant obtains the amount of $w_{b}$ and $w_{b+c}$ that the user continues to use; then, $w_{b+c}$ is used to calculate the hash function *c* times to determine whether it is equal to $w_{b}$.

In a transaction, two transaction verification messages, μ and β, are sent. μ is the verification message provided to the merchant with which the following verification is conducted:

1. $w_{b}$ and $w_{b+c}$ are used to calculate the correctness;
2. the issuer’s public key is employed to decrypt φ to obtain $w_{0}$, and it is checked whether $w_{b}$ can be calculated back to $w_{0}$ to verify that the obtained hash value is correct;
3. the merchant checks that the forced addition to counter by the user is less than the applied amount *n*;
4. the merchant checks that $ID_{M}$ is the merchant’s own ID.

The variable β is a verification message provided to the issuer to perform symmetric encryption by using the shared key $K_{\beta }$. The issuer verifies the contents of β by:

1. the issuer verifies the merchant identity $s_{v}$;
2. the issuer checks whether $ID_{M0}$ is in the merchant list *M*;
3. the issuer obtains the serial number of the secret factor of the hash chains $SN_{w}$ and $SN_{s}$ and checks the corresponding hash value secret factors $w_{n}$ and $s_{0}$;
4. the issuer checks that the derived $R_{lim}$ is the same offline certificate and credit quota that was supplied by the user.

### 2.2. Merchant’s Identity

When users purchase from multiple merchants, the merchants must be unable to deny the purchases. The identity of each merchant is calculated using each hash value in the hash chain, which is as follows:

(2) $$s_{i+1}=h\left(s_{i}\right),\forall i=0,1,\cdots ,n.$$

$s_{0}$ is a hash function secret factor representing the identity of a merchant. In the first transaction, the mobile phone calculates $s_{1}$ to represent the merchant participating in the transaction. Similarly, in the second transaction, $s_{2}$ is calculated to represent the participating merchant. The secret factor of this hash chain $s_{0}$ is only shared between the issuer and mobile phone; the merchant is unaware which hash value it belongs to. Finally, the $s_{n}$ generated after the *n*th transaction (first item of the reverse hash chain) is stored in the phone’s secure element, and the user cannot modify the hash value of the representative merchant.

### 2.3. Compatibility with the EMV Protocol

The method proposed in this study is based on EPMAR, in which an unused field (EXTERNAL AUTHENTICATION command) of the EMV [1] command parameter is used to add new messages without changing the order of the original protocol. In addition, the unused option field (reserved for future use [RFU] of GENERATE *AC*) in the EMV message transfer is used to transfer the parameters and certificates required for authentication to improve security of offline transactions.

To distinguish parts of messages, blue font is used to indicate the newly added message and execution calculation by referring to EPMAR as the benchmark, and a green box indicates that the newly added message uses a field that is not used by EMV or has been reserved in order to be compatible with the EMV protocol.

Regarding the items held by mobile phones, merchants, and issuers during initialization, in addition to what is already employed in EPMAR (e.g., credit card data $Data_{emv}$, credit card private key $SK_{emv}$, communication key TK, merchant’s certificate $Cert_{m}^{acq}$, shared key $K_{enc_{emv}}$, message authentication code $K_{mac_{emv}}$, and merchant public key $PK_{m}$), mobile phones and merchants hold a public key $PK_{iss}$ of the issuer, which can be used to decrypt an encrypted message after endorsement by the issuer. The issuer adds (1) the issuer’s own key, $K_{iss}$, which is used to generate a credit quota and the secret value of the merchant’s hash function; (2) the issuer’s private key, $SK_{iss}$, which is employed to endorse the credit quota; and (3) the credit card’s public key, $PK_{emv}$, which shares the essential parameters needed for the offline transaction stored in the secure element of the credit card.

### 2.4. Phase 3: Offline Certificate Application and Split Credit Quota Authorization

#### 2.4.1. Phase 3 Process Diagram

**Figure 4:**
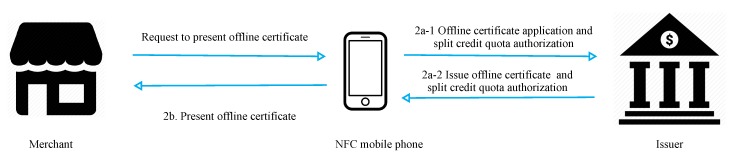
Offline certificate application and split credit line authorization processes.

Figure 4 presents a flowchart of offline certificate application and split credit quota authorization processes. In steps 1 and 2, the merchant requests an offline certificate from the user’s mobile phone, and the mobile phone applies to the issuer if it does not have an offline certificate.

Step 1: The merchant requests the user’s NFC phone to present its offline certificate.

Step 2: If the phone does not have an offline certificate,

- (2a-1) the phone applies to the issuer for a certificate and the split credit quota authorization required for transactions.
- (2a-2) the issuer generates the offline certificate, transaction authorization, and credit quota required to split the certificate. The certificate is stored in the secure element of the phone upon receipt.

If the phone has an offline certificate,

- (2b) the phone sends a message containing the offline certificate to the merchant.

#### 2.4.2. Phase 3 Protocol

**Figure 5:**
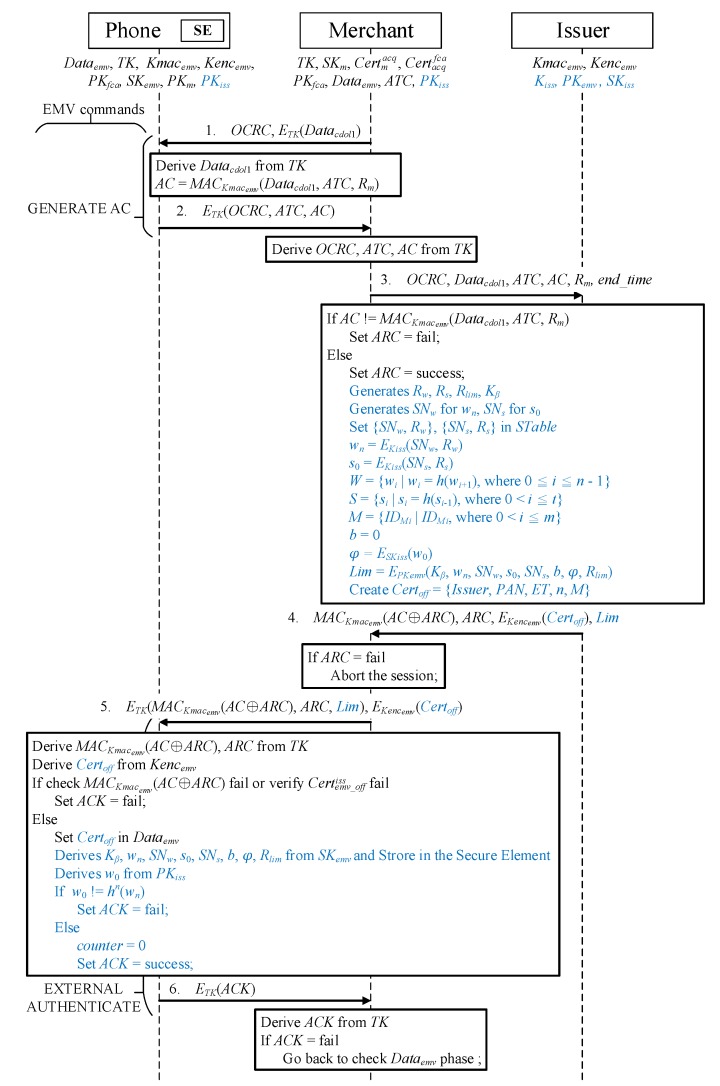
Offline certificate application and split credit line authorization protocol.

To make the protocol concise, the commands that a merchant sends to the credit card to obtain data types are omitted. The transmitted message is indicated by a solid arrow above, and the action performed after receiving the message is denoted by the solid box. A communication key, TK, is used to encrypt and protect the transaction between the phone and merchant’s POS. Figure 5 presents the protocol for offline certificate application.

- Message 1
  When the user’s phone receives a GENERATE *AC* command [1] containing an offline certificate request cryptogram (OCRC) [45], the phone first decrypts $E(Data_{cdol1})$ to retrieve $Data_{cdol1}$ by using the communication key TK. The data transferred by the merchant, $E(Data_{cdol1})$, serial number of the transaction, ATC [1], and a random number obtained in mutual authentication, $R_{m}$ [45], are used to generate a message authentication code with the hash function by using the shared key, $K_{mac_{emv}}$, with the issuer:
  (3) $$AC=MAC_{Kmac_{emv}}\left(Data_{cdol1},ATC,R_{m}\right).$$
- Message 2
  The phone encrypts the OCRC, ATC, and AC that were originally to be transmitted according to the protocol using TK and sends them back to the merchant to begin the offline certificate application phase.
- Message 3
  After receiving the message $E_{TK}\left(OCRC,ATC,AC\right)$ from the phone, the merchant decrypts OCRC, ATC, and AC by using TK. Subsequently, in addition to sending the decrypted data $Data_{cdol1}$ and the $R_{m}$ generated in mutual authentication to the issuer, the merchant informs the issuer of the longest offline duration to be used, end_time [45], so that the issuer can set the expiration time of the offline certificate according to the offline transaction environment.
- Message 4
  Once the issuer receives the OCRC request message for offline certificate application, it uses the shared key $K_{mac_{emv}}$, $Data_{cdol1}$, ATC, and $R_{m}$ in the message to calculate $MAC_{Kmac_{emv}}(Data_{cdol1},$$ATC,R_{m})$ and determine whether the AC code in the message sent by the merchant is correct. If the message is incorrect, the authorization response code (ARC) is set to fail. Otherwise, the ARC is set to success, and the issuer starts to generate the parameters and transaction verification message required for issuance of the offline transaction certificate and credit control:
  1. Randomly generated numbers $R_{w}$ and $R_{s}$ are used to create the corresponding serial numbers $SN_{w}$ and $SN_{s}$, and $\left\{SN_{w},R_{w}\right\}$ and $\left\{SN_{s},R_{s}\right\}$ are stored in STable.
  2. The $K_{iss}$ of the issuer is employed to encrypt the random and serial numbers to generate secret factors representing the credit quota $w_{n}$ and offline transaction merchant $s_{0}$:
    (4) $$w_{n}=E_{K_{iss}}\left(SN_{w},R_{w}\right),$$
    (5) $$s_{0}=E_{K_{iss}}\left(SN_{s},R_{s}\right).$$
  3. The hash values of $w_{n}$ and $s_{0}$ are calculated and placed into the reverse hash chain set *W* and forward hash chain set *S*:
    (6) $$W=\{w_{i}|w_{i}=h\left(w_{i+1}\right),0\leq i\leq n-1\},$$
    (7) $$S=\{s_{i}|s_{i}=h\left(s_{i-1}\right),0<i\leq t\}.$$
  4. All authorized merchant identities $ID_{M_{i}}$ are placed in the authorized merchant set *M*.
  5. A $K_{\beta }$ is generated that enables the issuer to authenticate the transaction message.
  6. A *b* is generated that represents the current user’s amount spent. Because the amount has not been used at the application phase, b=0.
  7. The issuer’s $SK_{iss}$ is used to perform asymmetric encryption $\varphi =E_{SK_{iss}}\left(w_{0}\right)$ on the last item $w_{0}$ of the credit quota hash chain for issuer endorsement. During the transaction, the merchant confirms the correctness of $w_{0}$ and calculates and verifies that the amount received is correct.
  8. Asymmetric encryption is performed using the credit card’s public key $PK_{emv}$, inserting the (a) shared key $K_{\beta }$ of the user and issuer; (b) secret factor $w_{n}$ of the limit hash chain; (c) serial number $SN_{w}$ of $w_{n}$; (d) $s_{0}$, which represents the secret factor of the merchant’s hash chain; (e) serial number $SN_{s}$ of $s_{0}$; (f) amount to be spent *b*; (g) φ after $w_{0}$ has been endorsed by the issuer; and (h) apart from necessary amount data, a random number $R_{lim}$, which prevents the amount spent message from being resent:
    (8) $$Lim=E_{PK_{emv}}\left(K_{\beta },w_{n},SN_{w},s_{0},SN_{s},b,\varphi ,R_{lim}\right).$$
  9. According to EMV standards, the end_time sent by the merchant, and the user’s credit assessment, the issuer generates $Cert_{off}$, an X.509 certificate [50]. This certificate includes the issuer (Issuer), user’s credit card account (PAN), offline certificate expiration time (ET), consumption upper limit (*n*), and all authorized merchant identities (*M*).
  Finally, the issuer uses $K_{mac_{emv}}$ to generate a message authentication code $MAC_{Kmac}(AC⊕$*ARC*) by using the mutually exclusive results between ARC and AC. Subsequently, this message authentication code, ARC, the $E(K_{enc_{emv}}\left(Cert_{off}\right)$ encrypted by the phone’s $K_{enc_{emv}}$, and Lim are sent to the merchant.
- Message 5
  If the merchant receives a reply from the issuer that the ARC fails, the offline certificate application process is terminated. However, a successful ARC indicates that the issuer agrees to send an offline certificate. The merchant uses the EXTERNAL AUTHENTICATION command marked in the unused parameter field [1] to transfer the offline certificate to the user’s mobile phone [45]. In addition, Lim is placed in this unused parameter field.
  Decryption and verification are performed after the phone has received $E_{TK}(MAC_{Kmac_{emv}}$(AC⊕*ARC*),*ARC*,Lim) and $E(K_{enc_{emv}}\left(Cert_{off}\right)$:
  1. Decryption is performed using TK, and the ARC received, AC calculated at the beginning of the protocol, and $K_{mac_{emv}}$ are used to calculate the message authentication code $MAC_{Kmac_{emv}}(AC⊕$*ARC*) to confirm that it is similar to the code received.
  2. $K_{enc_{emv}}$ is employed to decrypt the offline certificate $Cert_{off}$ and inspect its source. The phone sets the inspection result ARC to fail for a failed message authentication code and offline certificate verification.
  3. If the message received is verified to match the correct $Cert_{off}$, the certificate is placed in the $Data_{emv}$ list, making $Data_{emv}=Data_{emv}\cup Cert_{off}$.
  4. The $SK_{emv}$ of the credit card is used to decrypt the asymmetric encryption of Lim to obtain $K_{\beta }$, $w_{n}$, $SN_{w}$, $s_{0}$, $SN_{s}$, *b*, φ, and $R_{lim}$ and store them in the secure element for protection to prevent users from arbitrarily modifying the essential parameters and data of their offline transactions. Once the phone receives it, the Lim is sent directly to the secure element for decryption. The phone plays a mediating role between the merchant and secure element.
  5. The issuer’s public key is employed to decrypt the asymmetric encrypted φ to obtain the $w_{0}$ generated by the issuer, and whether $w_{0}$ is equal to the $w_{n}$ obtained by decrypting Lim after *n* hash function calculations is determined. If $w_{0}\neq h^{n}\left(w_{n}\right)$, the ARC fails; otherwise, the ARC is success, with counter=0. The forced added counter in the secure element adds up the amount of each purchase to the counter until the upper limit *n* is reached; reapplication is then required to continue making offline transactions.
- Message 6
  Finally, the phone returns the ARC to the merchant. If the ARC fails, the merchant must reapply for a certificate. Otherwise, the user’s phone saves the offline certificate, and the phone does not need to reapply for an offline certificate in the next transaction if the message received by the merchant contains $Data_{emv}$, which indicates that offline transaction can commence immediately.

### 2.5. Phase 4: Offline and Online Transactions

**Figure 6:**
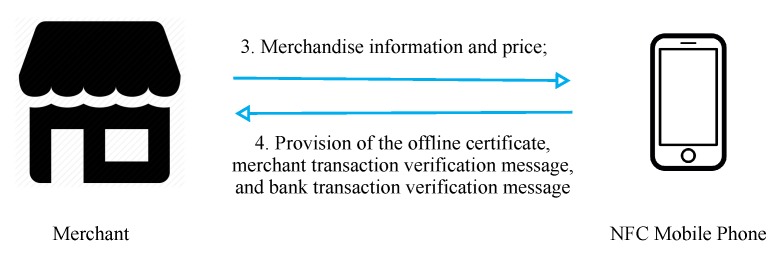
Offline transaction process.

Figure 6 presents a flowchart of offline transactions. In steps 3 and 4, the merchant provides relevant data to the user regarding purchased merchandise, and the user provides their certificate, payment, and verification information to the merchant upon confirmation.

In step 3, the user selects the merchandise to be purchased, and the phone displays the merchandise information and price for the user to confirm. In step 4, after the user has given their confirmation, the phone makes the payment and provides the merchant with the offline certificate and credit quota.

**Figure 7:**
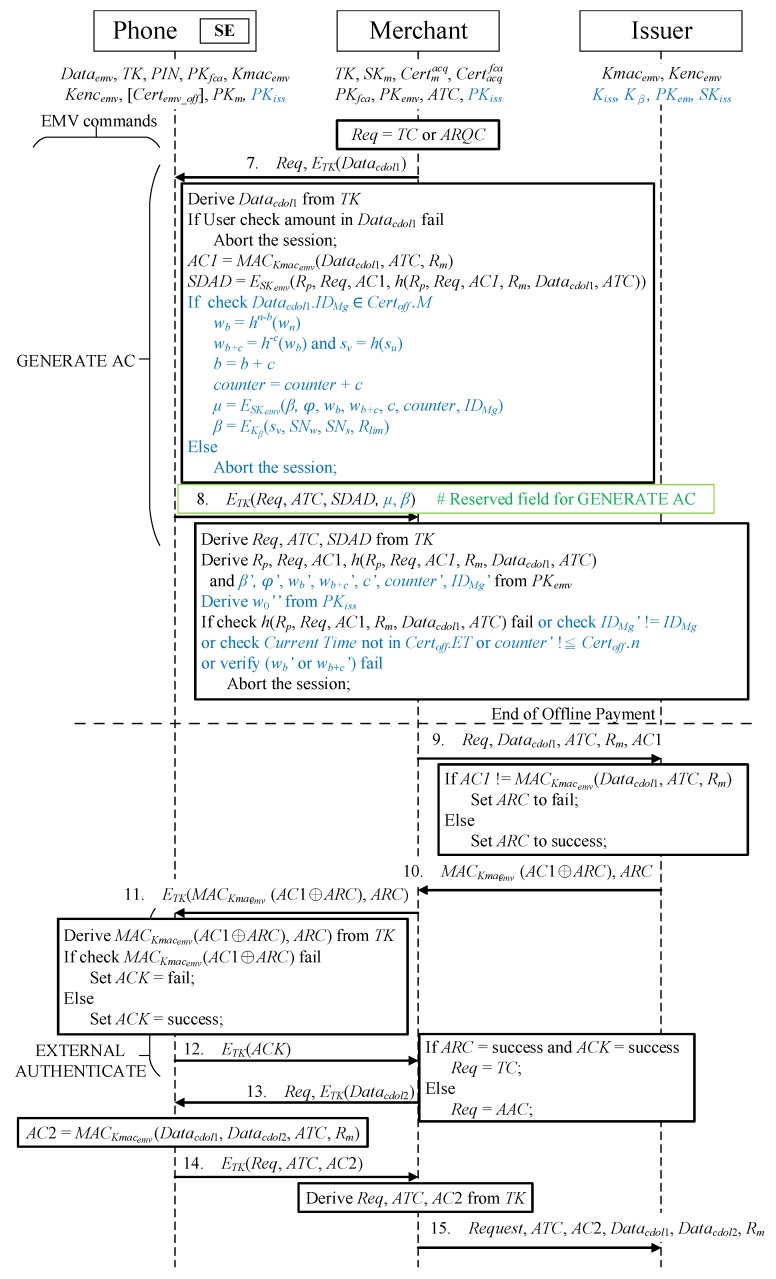
Offline/online transaction protocol.

In phase 4, the merchant transfers an offline/online transaction request (Req) and a GERNATE *AC* command (information required for transactions in the EMV protocol), encrypted by TK, to the mobile phone, as illustrated in Figure 7. If Req=ARQC, an online transaction is performed between the merchant and mobile phone; if Req=TC, an offline transaction is performed.

- Message 7
  Once the phone receives the GENERATE *AC* command with parameter Req, TK is used to decrypt the transaction data to obtain $Data_{cdol1}$, and the user confirms that the transaction amount in $Data_{cdol1}$ is correct. The phone then executes the following three steps in accordance with the EMV payment requirements:
  - A message authentication code is generated from the $Data_{cdol1}$, ATC, and $r_{m}$ using the issuer’s $K_{mac_{emv}}$. $AC1=MAC_{Kmac_{emv}}\left(Data_{cdol1},ATC,R_{m}\right)$.
  - $R_{p}$,$Data_{cdol1}$, ATC, the Req of AC1, AC1, and the $r_{m}$ for generating AC1 are calculated.
  - Encryption is performed using the EMV credit card’s $SK_{emv}$ to generate the signed dynamic application data (SDAD) from $R_{p}$, Req, AC1, and the hash function $h(R_{p},Req,AC1,$$R_{m},Data_{cdol1},ATC)$ generated in the previous step.
    The data stored in the secure element during the application phase are retrieved for verification and calculation, and transaction verification and payment messages are generated:
    1. The $ID_{M_{g}}$ sent by the merchant must be present in the authorized merchant list *M* of $Cert_{off}$; if it is not, the transaction is canceled immediately.
    2. The amount spent *b* is employed to calculate the used hash value $w_{b}=h^{n-b}\left(w_{n}\right)$. Moreover, the transaction amount c is used to calculate the paid hash value $w_{b+c}\;=\;h^{-c}\left(w_{b}\right)$. The amount spent is a reverse hash chain, and the hash is a one-way irreversible function; thus, the actual method for calculating the hash value is as follows:
      (9) $$w_{b+c}=h^{n-(b+c)}\left(w_{n}\right).$$
    3. The merchant code $s_{v}=h\left(s_{u}\right)$ is calculated. In the first purchase, u=0 and v=1. Each transaction generates a hash value $s_{i}$ representing the merchant, and the shared key is used for encryption so that the merchant is unaware of $s_{i}$; nonetheless, it does represent the merchant participating in the transaction.
    4. The amount *c* is spent in the transaction; thus, the amount spent becomes b=b+c.
    5. The current purchase amount *c* is added to counter, such that counter=counter+c.
    6. A verification message β is generated for the issuer, and the offline transaction authentication key $K_{\beta }$, which was obtained by the secure element during the application is used to represent the merchant’s identity hash value $s_{v}$, serial number $SN_{w}$ of $w_{n}$, serial number $SN_{s}$ of $s_{0}$, and $R_{lim}$ placed in Lim during the application phase; these are symmetrically encrypted to become $\beta =E_{K_{\beta }}\left(s_{v},SN_{w},SN_{s},R_{lim}\right)$.
    7. A verification message μ is generated for the merchant, and the $SK_{emv}$ of the credit card is employed to asymmetrically encrypt the issuer verification message β, amount after issuer endorsement φ, hash value of the amount spent $w_{b}$, hash value of the payment amount $w_{b+c}$, current amount spent *c*, offline transaction amount counter, and merchant’s identity $ID_{M_{g}}$ into $\mu =E_{SK_{emv}}\left(\beta ,\varphi ,w_{b},w_{b+c},c,counter,ID_{M_{g}}\right)$.
- Message 8
  The mobile phone encrypts Req, ATC, SDAD, μ, and β, which are returned to the merchant through the use of TK. The newly added message content is placed in the RFU of GENERATE *AC* for transfer to achieve an EMV-compatible protocol.
  The merchant receives the returned message $E_{TK}\left(Req,ATC,SDAD,\mu ,\beta \right)$ from the mobile phone and performs two decryption and six verification actions:
  - TK is used to decrypt $E_{TK}\left(Req,ATC,SDAD,\mu ,\beta \right)$ and extract Req, ATC, SDAD, μ, and β.
  - $PK_{emv}$ is employed to decrypt μ and obtain $\beta^{\prime },\varphi^{\prime },w_{b}^{\prime },w_{b+c}^{\prime },c^{\prime },{counter}^{\prime }$, and $ID_{M_{g}}$’, and the issuer’s public key is used to decrypt φ to obtain $w_{0}^{\prime \prime }$:
    1. The SDAD is decrypted using $PK_{emv}$, and the hash function $h(R_{p},Req,AC1,R_{m},$$Data_{cdol1},ATC)$ is verified.
    2. $ID_{M_{g}}$’ in μ is equal to $ID_{M_{g}}$.
    3. Whether current time meets the ET limited by the $Cert_{off}$ is checked.
    4. Whether the counter limit has not exceeded the offline transaction amount *n* set in the offline certificate is checked.
    5. The correctness of $w_{b}^{\prime }$ is calculated. Using $w_{b}^{\prime }=h^{-i}\left(w_{0}^{\prime \prime }\right)$, where 0≤i≤n, it is determined whether $w_{b}^{\prime }$ and $w_{0}^{\prime \prime }$ are in the same hash chain. Whether $w_{b+c}^{\prime }$ is equal to $w_{b}^{\prime }$ after *c* hash function calculations is determined, and the calculation method is $h^{c^{\prime }}\left(w_{b+c}^{\prime }\right)=w_{b}^{\prime }$.

The transaction is terminated immediately if any verification fails. Finally, receipt of Req=TC indicates that the transaction is an offline transaction, and the merchant requests payment from the issuer to complete the transaction. However, if Req=ARQC is received, the merchant transfers data to the issuer to begin an online transaction. The transfer content of the online transaction begins from Message 3.

### 2.6. Phase 5: Payment Request by the Merchant

**Figure 8:**
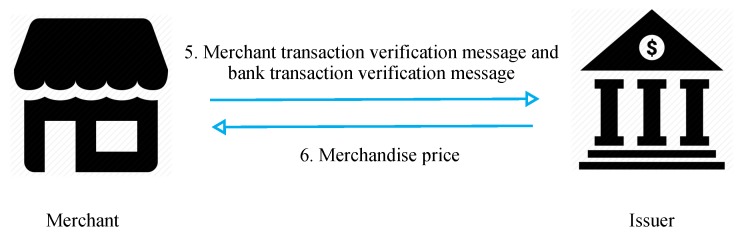
Process of merchant payment request.

Figure 8 presents the merchant payment request diagram. In steps 5 and 6, the merchant transfers the payment information and verification message to the issuer to verify the correctness of the transaction. If the information is correct, the issuer sends the amount corresponding to the user’s purchase to the merchant.

- Step 5: the merchant sends the self-verified message, issuer-verified message, and payment information to the issuer to request verification of the transaction’s correctness.
- Step 6: if the transaction is verified, the merchant can request payment from the issuer.

**Figure 9:**
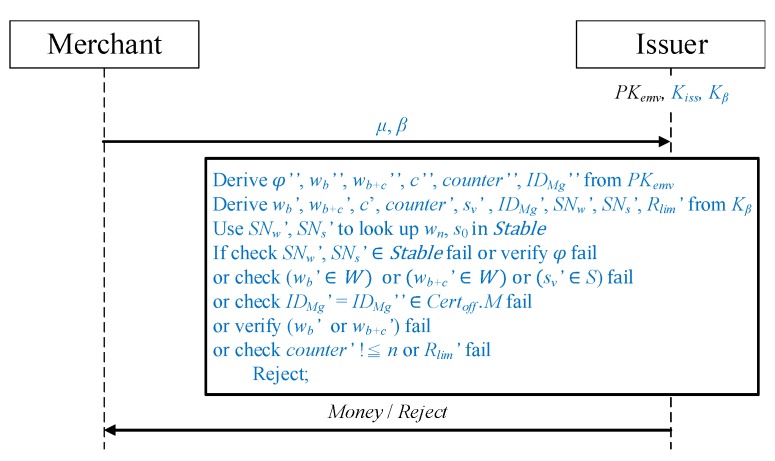
Merchant payment request protocol.

Once the offline transaction is complete, the merchant sends the payment message received to the issuer. As illustrated in Figure 9, in addition to the transaction message of a payment request, an additional verification required by the issuer (in the method proposed in this study) is added.

The merchant sends the verification messages μ and β to the issuer. The issuer decrypts the messages using $K_{\beta }$ and $PK_{emv}$, respectively, to obtain $\left\{sv^{\prime },SN_{w}^{\prime },SN_{s}^{\prime },R_{lim}^{\prime }\right\}=D_{K_{\beta }}\left(\beta \right)$ and $\left\{\beta^{\prime \prime },\varphi^{\prime \prime },w_{b}^{\prime \prime },w_{b+c}^{\prime \prime },c^{\prime \prime },{counter}^{\prime \prime },ID_{M_{g}}^{\prime \prime }\right\}=D_{PK_{emv}}\left(\mu \right)$.

Whether $SN_{w}^{\prime }$ and $SN_{s}$′ in β exist in STable is checked. If they do, sequence numbers $SN_{w}$ and $SN_{s}$ are used to obtain the random numbers $R_{w}$ and $R_{s}$ from the table. Subsequently, $\left\{SN_{w},R_{w}\right\}$, $\left\{SN_{s},R_{s}\right\}$, and the issuer’s $K_{iss}$ are employed to perform encryption for calculating $w_{n}=E_{K_{iss}}\left(SN_{w},R_{w}\right)$ and $s_{0}=E_{K_{iss}}\left(SN_{s},R_{s}\right)$ to obtain the $w_{n}$ and $s_{0}$ applied by the user. After obtaining $w_{n}$ and $s_{0}$, the issuer makes checks and verifications to determine whether the transaction amount should be issued to the merchant:

1. It is verified that $w_{b}$ and $w_{b+c}$ are in the *W* hash value set generated during the application phase and the merchant representative hash value $s_{v}$ is in the *S* hash value set.
2. Whether $ID_{M_{g}}$ in μ is present in the list of authorized merchants of the offline certificate $Cert_{off}$ is determined.
3. The preposition $w_{b}^{\prime }=h^{-i}\left(w_{0}\right)$, where 0≤i≤n, is considered to determine whether $w_{b}^{\prime }$ and $w_{0}$ are in the same hash chain and thus confirm their correctness. Whether $w_{b+c}^{\prime }$ equals $w_{b}^{\prime }$ after *c* hash function operations is determined, and its calculation method is $h^{c^{\prime }}\left(w_{b+c}^{\prime }\right)=w_{b}^{\prime }$.
4. Whether counter exceeds the limit *n* is checked.
5. It is confirmed that the $R_{lim}$ in β is equal to the $R_{lim}$ placed in Lim during the application phase.

If the aforementioned verification fails and the comparison result does not conform to the issuer, the transaction is rejected and reviewed. If the verification is successful, the issuer pays the amount to the merchant.

## 3. Security Analysis and Performance Evaluation

The security of the method proposed in this study and the performance are analyzed in this chapter.

### 3.1. Security Analysis

1. Verifiability:
  - In phase 4, the merchant can immediately verify the verification message of the offline transaction.
  - In phase 5, the issuer obtains transaction verification messages (for the merchant and issuer) from the merchant and verifies all the information obtained to ensure the correctness of the transaction.
2. Counterfeiting prevention:
  - In phase 3 (2a-2), the issuer provides the user and merchant with the encrypted hash value of the final item of the user’s credit quota chain $w_{n}$ so that they can verify the amount of the hash value (as shown in Figure 4). Attackers cannot encrypt the cipher text without the issuer’s private key.
  - In message 7 of phase 4, the amount spent *b* information given to the merchant is protecvalue $w_{b}=h^{n-b}\left(w_{n}\right)$ by the user is asymmetrically encrypted using the credit card private key $SK_{emv}$: $\mu =E_{SK_{emv}}\left(\beta ,\varphi ,w_{b},w_{b+c},c,counter,ID_{M_{g}}\right)$, and the merchant requests payment from the issuer after confirmation. Incorrect amount information generated by the user is immediately detected by the merchant. Similarly, the merchant cannot falsify the information given by the user to request a higher amount from the issuer.
3. Tampering prevention:
  - In the application phase, the offline transaction certificate and credit quota limit information $Lim=E_{PK_{emv}}\left(K_{\beta },w_{n},SN_{w},s_{0},SN_{s},b,\varphi ,R_{lim}\right)$ are encrypted using the virtual credit card’s public key $PK_{emv}$ and the key $K_{\beta }$ shared between the user and issuer, enabling the issuer to protect the message content. In addition to the credit quota hash chain’s secret factor $s_{0}$, the credit quota message contains cipher text encrypted using the issuer’s private key.
  - If the secret factor $s_{0}$ is not calculated by the original issuer, different hash values obtained during the verification will cause failure of the verification.
  - The verification message given to the merchant SDAD in message 8 of phase 4 is encrypted using the virtual credit card’s private key $SK_{emv}$, which is stored in the secure element. Even if malicious software is installed on the user’s mobile phone, the content encrypted by the secure element is not easily modified.
4. Replay attack prevention:
  - In the application phase, the issuer places a random number $w_{n}=E_{K_{iss}}\left(SN_{w},R_{w}\right)$ in the credit quota message and passes it to the user. The user places the random number in the issuer verification message (as shown in Figure 9 and passes it to the merchant during a transaction. The merchant sends this message to the issuer during their payment request. The issuer then checks whether the random number is the same as that given by the issuer to the user during application and whether the corresponding amount is the amount originally given as the hash value.
5. Nonrepudiation:
  - In phase 4, in addition to having a transaction message, the user has a verification message (to be given to the merchant) that is encrypted using the virtual credit card’s private key $SK_{emv}$ to generate the signed dynamic application data (SDAD) from $R_{p}$, Req, AC1, which ensure that the message is sent by the user. The user cannot deny that they have created this message.
  - The issuer verification message $\mu =E_{SK_{emv}}\left(\beta ,\varphi ,w_{b},w_{b+c},c,counter,ID_{M_{g}}\right)$ also contains two corresponding hash values ($w_{b}$ and $w_{b+c}$) for the transaction that the merchant is not informed of, indicating that the credit quota is for the use of a certain merchant. If the merchant subsequently denies the transaction, the issuer can identify the corresponding merchant during verification.
6. Duplicate payment prevention:
  - The user cannot cheat the merchant because a counter is stored in the secure element of the mobile phone and is forced to increase upon every transaction. The value of counter must be smaller than the transaction usage amount requested by the user. If the user repeatedly uses the amount of the hash value, the counter is still forcibly added to; thus, the amount spent must be lower than the applied amount (e.g., a failed transaction may waste the usable amount because the counter has already been forcibly added to when the user sends out a credit quota limit hash value), and the amount does not expand because of a duplicate payment. If the merchant want to duplicate the payment (as shown in Figure 8), it sends μ and β to the issuer. The issuer will decrypt the message and detect double-spending by using the $w_{b}$ and $w_{b+c}$ are in the *W* hash value set generated during the application phase.

Table 2 shows the security comparison between our protocol EOPMA, EPMAR [45], and the original EMV standards [1]. We also compare our protocol with Al-Tamimi [28] and Madhoun [29] works. EOPMA, EPMAR and Modhoun’s scheme perform mutual authentication with the merchant. Both of them can detect a malicious phone and a malicious merchant. The original EMV standards (CDA, DDA and SDA) only authenticate with the phone, which causes an MITM attack to possibly occur. In addition, in an EMV transaction, the data between a reader and a card are transmitted in plaintext.

In Al-Tamimi’s scheme, it adds a Mobile Network Operator (MNO) layer as a trust third party to perform mutual authentication between the NFC phone and the merchant. However, it requires extra communication channel, which is not fully EMV-compatible.

### 3.2. GNY Logic Proof

In this section, we use the Gong–Needham–Yahalom (GNY) logic [51] to prove the security of our proposed protocol. The GNY logic is used for the analysis of cryptographic protocols in a formal way, which can be easily applied and gives a quick insight in the working of a protocol. Our analysis includes four parts: the notation of the GNY proof (Table 3), initial assumptions (Table 4), goals of proposed protocol (Table 5), and the proving process (Table 6).

### 3.3. Performance Analysis

In the current EMV standard, the asymmetric encryption uses RSA 1024 bits, we also add RSA 2048 bits as the object of analysis. In symmetric encryption, we chose AES-128 as the benchmark for our symmetric encryption. In order to compare with the original EMV standards, we chose the same experimental environment as EPMAR; use two E975 LG Optimus G mobile phones at Taiwan to negotiate the consumer side and the merchant side, and use the Android API level 16 library to implement EOPMA. The transmission rate between the phone and the reader is 858 kbit/s, according to ISO 14443 standard. Table 7 lists the operation time of cryptography functions of the LG mobile phone. Due to the restriction of EMV standards, an EMV transaction should take less than 500 ms, we choose RSA-1024 in our protocol evaluation.

Next, we compare the extra computational loads with EPMAR. We assume the computation capabilities of the issuer are better than the mobile phone and the merchant’s POS. Table 8 shows the extra operation time of EOPMA, comparing to EPMAR. We use the symbol $T_{H}$, $T_{A}$, $T_{RSA_{E}}$, $T_{RSA_{D}}$, T−r as HMAC, AES, RSA encryption, RSA decryption and random number generation, respectively. In phase 1 and phase 2, the EOPMA operations are the same as EPMAR. In phase 3, the issuer requires extra six random numbers’ generation, plus two hash chains (*W* and *S*) calculations. The *W* hash chain is for the credit quota generation and the *S* hash chain is used to represent the count of merchants. The phone needs an AES decryption and *n* HMAC-128 to calculate the hash chain $w_{n}$ (n∗H).

In phase 4, the phone only needs one AES encryption and one RSA encryption. The merchant requires one AES decryption, one RSA decryption and up to (c-b) hash operations. In phase 5, the merchant only forwards the payment information to the issuer; all the verifications are done in the issuer.

**Figure 10:**
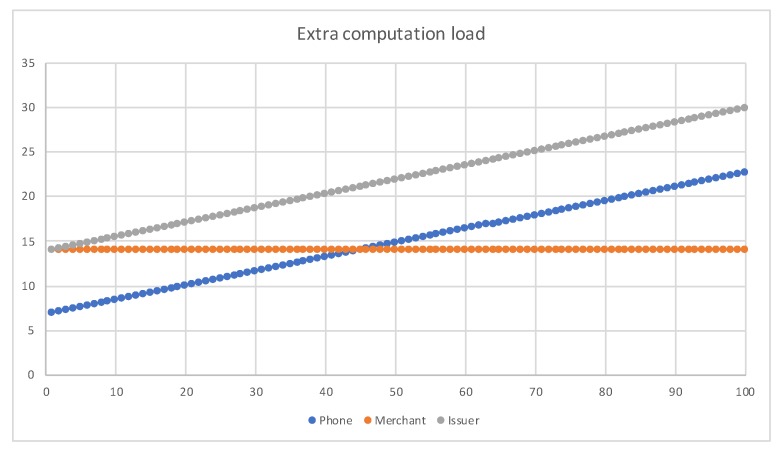
Extra computation time.

In the experiment, we choose n=1..100, for the maximum of 100 offline transactions, and the maximum offline transactions per merchant, (c−b)=10. The result is shown in Figure 10. The extra spent time of the phone is less than 23 ms. The issuer requires 29.74 ms for 100 transactions. However, the computing power of a server far exceeds that of a mobile phone. The issuer’s calculation time will be smaller than the experiment.

## 4. Conclusions

In this paper, we proposed an offline mobile payment protocol named EOPMA that is compatible with EMV, provides mutual authentication, and can solve the problems of credit quota exceeding in EPMAR and duplicate payments in PayWord. In EOPMA, an offline transaction is halted when the offline credit quota reaches the amount specified by the user, and the user must then reapply to the issuer to make further purchases. Through the proposed offline certificate and credit control, both the issuer and merchant can clearly verify the content of transaction information and the credit quota given by users, protecting them from losses incurred. Using EOPMA, the amount spent will never exceed the credit quota imposed by the issuer to ensure credit control and solve the double-spending problem.

We implemented EOPMA on an NFC phone and compared the performance with other schemes. EOPMA implemented EMV’s unused and reserved commands to add a new method that is compatible with the existing EMV protocol. Our protocol resists the security threats in the EMV standards, such as man-in-the-middle attacks, replay attacks, and clone attacks. The cryptographic protocol analysis logic of Gong, Needham and Yahalom (GNY) is used to prove the correctness of EPMAR. 

## Figures and Tables

**Table 1:** Sequence numbers and random numbers of secret factors.

Serial Number	Random Nonce
⋯	⋯
$SN_{w}$	$R_{w}$
⋯	⋯
$SN_{s}$	$R_{s}$

**Table 2:** Security comparison chart.

	*EOPMA*	*EPMAR*	Al-Tamimi	Modhoun	*EMV-CDA*	*EMV-DDA*	*EMV-SDA*
Exceeding of a credit quota	O	X	X	X	X	X	X
Malicous phone	O	O	O	O	O	O	O
Malicous merchant	O	O	O	O	X	X	X
Confidentiality	O	O	O	O	X	X	X
Replay attacks	O	O	O	O	X	X	X
Data privacy	O	O	O	O	X	X	X
Integrity	O	O	O	O	O	O	O
Non-repudiation	O	O	O	O	O	X	X
MITM attacks	O	O	X	O	O	X	X
Clone attacks	O	O	O	O	X	X	X
Fully EMV-Compatiable	O	O	X	O	O	O	O

**Table 3:** Notations of the GNY proof.

I	Issuer
M	Merchant
P	User’s NFC phone
${\left\{X\right\}}_{K}$,${\left\{X\right\}}_{K}^{-1}$	Uses the symmetric key *K* to encrypt/decrypt the message *X*.
${\left\{X\right\}}_{+K}$,${\left\{X\right\}}_{-K}$	Uses the asymmetric key *K* to encrypt/decrypt the message *X*.
H(X)	Message *X* is protected by the one way hash function H(X).
P◃X	*P* is told message *X*.
P∈X	*P* possesses message *X*.
∗X	*X* is generated by others; P◃∗X means *P* is told for *X* which he did not convey previously.
P∣≡#(X)	*P* believes *X* is fresh. *X* has not been used at any time in the prior protocol, or sent by an attacker.
	For example, a random number or a counter.
P∣≡⌀(X)	*P* believes *X* is recognizable.
P∣≡PSQ	*P* believes *S* is shared by *P* and *Q*.
P∣≡P+KQ	*P* believes *Q* owns the private key −K correspondent to the public key +K.
P∣≡Q∣∼X	*P* believes *Q* sent *X*.

**Table 4:** Initial assumptions.

Phone	
$P\in -K_{emv},+K_{emv},Kenc_{emv},TK$	The phone keeps a virtual credit card’s public key, private key,
$P|\equiv PKenc_{emv}I$	and the shared encryption key.
P∣≡PTKM	The phone generates a session key TK with a merchant during a transaction.
$P|\equiv \overset{+K_{iss}}{\to }I$	The phone holds the issuer’s public key.
$P|\equiv ⌀(ID_{M_{g}})$	The phone knows the merchant’s ID.
Merchant	
$M\in ID_{M_{g}},TK$	The merchant owns his identity,
M∣≡MTKP	the shared session key TK with the phone,
$M|\equiv M+K_{emv}P$	credit card’s public key,
$M|\equiv M+K_{iss}P$	and the issuer’s public key.
Issuer	
$I\in -K_{iss},+K_{iss},Kenc_{emv},K_{\beta }$	The issuer has its own private key and public key,
$I|\equiv IKenc_{emv}P$	the shared key with the phone,
$I|\equiv \overset{+Kenc_{emv}}{\to }P$	the transaction key.
$I|\equiv ⌀(ID_{M_{g}})$	The issuer knows the merchant’s identity,
$I|\equiv \#(R_{lim})$	and uses a random number for verification.

**Table 5:** Goals of proposed protocol.

Phase 3, offline certificate application and split credit quota authorization	
$P|\equiv I|∼\#(R_{lim})$	The phone believes $R_{lim}$ and $Cert_{off}$ is generated by the issuer,
$P|\equiv I|\equiv ⌀\#(R_{lim})$	gets the shared key $K_{\beta }$.
$P|\equiv I∼\#(K_{\beta },w_{n},SN_{w},s_{o},b,\varphi ,R_{lim})$	Finally both of them believes all the messages are fresh
$P|\equiv ⌀(K_{\beta },w_{n},SN_{w},s_{o},b,\varphi ,R_{lim})$	and are recognizable.
$P|\equiv PK_{\beta }I$	
$P|\equiv I|∼Cert_{off}$	
$P|\equiv I|⌀Cert_{off}$	
Phase 4, offline and online transactions	
$M|\equiv P|⌀Cert_{off}$	The merchant believes the phone has the offline certificate $Cert_{off}$,
M∣≡P∣∼(μ)	and he believes μ is generated by the phone.
M∣≡P∣⌀(μ)	Finally the two parties believe the μ is fresh
$M|\equiv P|∼\#(\beta ,\varphi ,w_{b},w_{b+c},c,counter,ID_{M_{g}})$	and is recognizable.
$M|\equiv ⌀(\beta ,\varphi ,w_{b},w_{b+c},c,counter,ID_{M_{g}})$	
Phase 5, payment request by merchant	
I∣≡M∣∼⌀(μ)	Both the issuer and the merchant believe μ is recognizable,
$I|\equiv M|⌀(\beta ,\varphi ,w_{b},w_{b+c},c,counter,ID_{M_{g}})$	and the issuer believes β is recognizable.
I∣≡⌀(β)	
$I|\equiv ⌀(s_{v},SN_{w},SN_{s},R_{lim})$	

**Table 6:** Proving process.

Phase 3		
Message	$M◃*R_{lim},*{\left\{Cert_{off}\right\}}_{Kenc_{emv}}$	The merchant and the issuer has built an secure channel
1.1	$M|\equiv I|∼R_{lim},*{\left\{Cert_{off}\right\}}_{Kenc_{emv}}$ /* IA */	all the messages between them can be trusted.
	$M|\equiv \#(R_{lim},{\left\{Cert_{off}\right\}}_{Kenc_{emv}})$ /* IA */	
	$M|\equiv ⌀(R_{lim},{\left\{Cert_{off}\right\}}_{Kenc_{emv}})$ /* IA */	
Message	$P◃*{\left\{R_{lim}\right\}}_{TK},*{\left\{Cert_{off}\right\}}_{Kenc_{emv}}$	The phone believes TK is fresh.
1.2	$P◃{\left\{R_{lim}\right\}}_{TK},{\left\{Cert_{off}\right\}}_{Kenc_{emv}}$ /*T1*/	believes μ is generated by the phone.
	$P◃R_{lim},Cert_{off}$ /* T3 */	Finally both of them believes the μ is fresh
	$P\in R_{lim},Cert_{off}$ /* P1 */	and is recognizable.
	$P|\equiv \#(R_{lim})$ /* F3 */	
	$P|⌀\#(R_{lim})$ /* R4 */	
	$P|\equiv \#(Cert_{off})$ /* F3 */	
	$P|⌀\#(Cert_{off})$ /* R4 */	
	$P◃K_{\beta },w_{n},SN_{w},s_{0},b,\varphi ,R_{lim}$ /* T3 */	
	$P\in K_{\beta },w_{n},SN_{w},s_{0},b,\varphi ,R_{lim}$ /* P1 */	
	$P|\equiv ⌀(K_{\beta },w_{n},SN_{w},s_{0},b,\varphi ,R_{lim})$	
	$P|\equiv PK_{\beta }I$ /* J1 */	
Phase 4		
Message	$P◃*Req,*{\left\{Data_{emv}\right\}}_{TK}$	The phone believes TK is fresh,
2.1	$P◃Req,{\left\{Data_{emv}\right\}}_{TK}$ /* T1 */	and $Data_{cdol1}$ is recognizable.
	$P◃Data_{cdol1}$ /* T3 */	Therefore, $Data_{cdol1}$ is not forged or replayed.
	$P\in Data_{cdol1}$ /* P1 */	
	$P|\equiv ⌀Data_{cdol1}$ /* R2 */	
Message	$M◃*{\left\{\mu \right\}}_{TK}$	The merchant believes TK is fresh, and μ is not forged.
2.2	$M◃{\left\{\mu \right\}}_{TK}$ /* T1 */	The merchant gets the public key $+K_{emv}$ of the credit card.
	M◃μ /* T3 */	Therefore, the merchant can vertify the message μ by φ.
	M∈μ /* P1 */	
	M∣≡#(μ) /* F4 */	
	$M◃\beta ,\varphi ,w_{b},w_{b+c},c,counter,ID_{M_{g}}$ /* T6 */	
	$M\in \beta ,\varphi ,w_{b},w_{b+c},c,counter,ID_{M_{g}}$ /* P1 */	
	$M|\equiv ⌀(\beta ,\varphi ,w_{b},w_{b+c},c,counter,ID_{M_{g}}$	
	M∣≡⌀(φ) /* R3*/	
	$M◃w_{0}$ /* T6 */	
	$M\in w_{0}$/* T6 */	
	$M|\equiv w_{0}$ /* P1 */	
Message	I◃∗μ	The issuer and the merchant has established a secure channel,
3	I◃μ /* IA, T1*/	all the messages exchanged between them can be trusted.
	I∣≡M∣∼μ/∗IA∗/	The issuer gets phone’s public key $+K_{emv}$, and the shared
	I∈μ/∗P1∗/	key $K_{\beta }$.
	I∣≡#(μ) /* IA, F4 */	Therefore, the issuer can verify the message μ and β.
	I∣≡⌀(μ). /* IA, R3 */	
	$I◃\beta ,\varphi ,w_{b},w_{b+c},c,counter,ID_{M_{g}}$ /* T6 */	
	$I\in \beta ,\varphi ,w_{b},w_{b+c},c,counter,ID_{M_{g}}$ /* P1*/	
	$I\in \equiv ⌀(w_{b},w_{b+c},c,counter,ID_{M_{g}}$	
	I∣≡#(β) /* F2 */	
	I∣≡⌀(β) /* R2 */	
	I∣≡⌀(φ) /* R3*/	
	$I◃s_{v},SN_{w},SN_{s},R_{lim}$ /* T3 */	
	$I◃s_{v},SN_{w},SN_{s},R_{lim}$ /* T3 */	
	$I\in s_{v},SN_{w},SN_{s},R_{lim}$ /* T3 */	
	$I\in ⌀(s_{v},SN_{w},SN_{s},R_{lim})$	

**Table 7:** The operation time of cryptography functions (ms).

*Operations*	Time
AES-128 ($T_{A}$)	0.15
HMAC-128 ($T_{H}$)	0.16
HMAC-256 ($T_{H_{256}}$	0.25
Random number generation	0.01
RSA encrypt ($T_{RSA_{E}}$)	0.59
RSA decrypt ($T_{RSA_{D}}$)	5.67

**Table 8:** The extra operations of EOPMA, comparing to EPMAR.

*Operations*	Phone	Merchant	Issuer
Phase 1	0	0	0
Phase 2	0	0	0
Phase 3	$T_{A}+n*T_{H}+T_{RSA_{D}}$	$T_{A}+T_{RSA_{E}}+T_{RSA_{D}}+T_{H}$	$6*T_{r}+n*T_{H}+T_{RSA_{E}}+T_{RSA_{D}}$
Phase 4	$T_{A}+T_{RSA_{E}}$	$\left(c-b\right)*T_{H}+T_{A}+T_{RSA_{D}}$	0
Phase 5	0	0	$T_{A}+T_{RSA_{D}}+\left(c-b\right)*T_{H}$

## Notations

**Symbols**	**Definitions**
$Cert_{target}^{publisher}$	Publisher signs the certificate issued to the target, and the certificate contains a public key corresponding to the target’s private key.
$Cert_{off}$	Required certificate for offline transactions.
$PK_{target}$	Target’s public key.
$SK_{target}$	Target’s private key.
$Kenc_{emv}$	A symmetric encryption key shared between the credit card and issuer.
$Kmac_{emv}$	A symmetric key shared between the credit card and issuer for calculating the message authentication code.
$K_{\beta }$	A symmetric key shared between the credit card and issuer for encrypting the offline transaction certificate message provided to the issuer.
TK	A communication key for the mobile phone and merchant.
$K_{iss}$	A key owned by the issuer for encryption of specific serial numbers and random numbers to generate secret values for the hash function chain.
$E_{key}\left(M\right)$	Encryption of message *M* by using a symmetric or asymmetric key.
$D_{key}\left(M\right)$	Decryption of message *M* by using a symmetric or asymmetric key.
$MAC_{Kmac_{emv}}\left(M\right)$	In the EMV protocol, a symmetric authentication function and a key $Kmac_{emv}$ are used to calculate the message authentication code function of message M.
h(M)	The hash function and *key* are employed to calculate the message authentication code function of message *M*.
*n*	The maximum credit quota during an offline transaction, which also represents the number of hash functions that can be calculated by the hash function chain of the credit quota.
*t*	Maximum number of offline transactions.
*m*	Number of merchants authorized for offline transactions.
$w_{n}$	The first item of the hash function chain, which can perform *n*th hash function calculations for the limit of offline transactions.
$w_{0}$	The last item in the hash function chain for the credit quota for offline transactions.
$s_{0}$	The first item of the hash function chain, which is used to represent the secret factor of the merchant participating in the offline transaction.
$s_{n}$	The *n*th item of the hash function chain, which is used to represent the merchant participating in the offline transaction, indicating the merchant corresponding to the *n*th offline transaction.
$i^{\prime }$, $i^{\prime \prime }$	The plaintext *i* obtained after decryption using the key.
STable	The table in which the issuer places the $w_{n}$ and $s_{0}$ elements.
$SN_{i}$	The serial number corresponding to *i*.
$R_{i}$	The random number used for *i*.
$R_{lim}$	The random number attached to the credit quota issuance.
Lim	The required content for offline transaction amount management and restrictions.
*W*	A set of hash values for all amounts.
*S*	A set of hash values for all corresponding merchants.
*M*	A set of all merchants authorized to complete offline transactions.
$ID_{M}$	The identification name of merchant *i*.
counter	Offline transaction amount counter.
φ	The credit quota issued by the issuer.
*l*	The number of calculations of the hash function corresponding to this consumption amount.
$end_{t}ime$	The longest offline transaction duration notified by merchants.
PAN	Primary account number, which is the user’s credit card account number.
ET	Expiry time, which is the effective time taken to complete the offline transaction.

## References

[1] (2011). EMVCo: EMV—Integrated Circuit Card Specifications for Payment System.

[2] (2014). EMVCo: A Guide to EMV Chip Technology.

[3] De Ruiter J., Poll E. Formal Analysis of the EMV Protocol Suite. Proceedings of the 2011 international conference on Theory of Security and Applications (TOSCA 2011).

[4] Chen C., Tang S., Mitchell C.J. Building General-Purpose Security Services on EMV Payment Cards. Proceedings of the 8th International Conference on Security and Privacy in Communication Networks.

[5] Murdoch S.J., Anderson R. Security Protocols and Evidence: Where Many Payment Systems Fail. Proceedings of the 8th International Conference on Financial Cryptography and Data Security.

[6] Alhothaily A., Alrawais A., Cheng X., Bie R. Towards More Secure Cardholder Verification in Payment System. Proceedings of the 9th International Conference on Wireless Algorithms, Systems, and Applications (WASA).

[7] (2013). ECMA INTERNATIONAL: Standard ECMA-340, Near Field Communication Interface and Protocol (NFCIP-1).

[8] (2013). ECMA INTERNATIONAL: Standard ECMA-352, Standard ECMA-340, Near Field Communication Interface and Protocol -2 (NFCIP-2).

[9] (2013). Information Technology—Telecommunications and Information Exchange between Systems—Near Field Communication Interface and Protocol-1 (NFCIP-1).

[10] (2012). Information Technology—Telecommunications and Information Exchange Between Systems—Near Field Communication Interface and Protocol-2 (NFCIP-2).

[11] (2008). Identification Cards—Contactless Integrated Circuit Cards—Proximity Cards—Part 1–Part 4.

[12] Giese D., Liu K., Sun M., Syed T., Zhang L. (2019). Security Analysis of Near-Field Communication (NFC) Payments. arXiv.

[13] (2009). Visa payWave—Visa Contactless Payment Specification (VCPS) Version 2.1.

[14] Steffens E.-J., Nennker A., Ren Z., Yin M., Schneider L. The SIM-based Mobile Wallet. Proceedings of the 13th International Conference on Intelligence in Next, Generation Networks (ICIN).

[15] Cheng H.C., Chen J.W., Chi T.Y., Chen P.H. A Generic Model for NFC-based Mobile Commerce. Proceedings of the 11th International Conference on Advanced Communication Technology.

[16] Noh S.K., Choi D.Y., Kim H.G., Kim D.K., Seo J.H., Kim J.W., Cha B.R. (2013). Proposed of Micropayment and Credit Card Model using NFC Technology in Mobile Environment. Int. J. Multimedia Ubiquitous Eng..

[17] Google Corp Google Wallet. http://www.google.com/wallet/.

[18] Microsoft Corp Trusted Platform Module (TPM) Virtual Smart Card Management Protocol Specification. http://msdn.microsoft.com/en-us/library/hh880895(prot.20).aspx.

[19] Apple Pay, Apple Inc. https://www.apple.com/apple-pay/.

[20] (2014). UL’s Independent Assessment, “Apple Pay—What Do We Know?”, White Paper. https://www.ul-ts.com/catalog/offerings/knowledge-sharing/whitepapers-and-case-studies/landing/c-29/c-1684.

[21] Pasquet M., Reynaud J., Rosenberger C. Secure Payment with NFC Mobile Phone in the SmartTouch Project. Proceedings of the International Symposium on Collaborative Technologies and Systems (CTS).

[22] Paillès J.C., Gaber C., Alimi V., Pasquet M. Payment and Privacy: A Key for the Development of NFC Mobile. Proceedings of the 2010 International Symposium on Collaborative Technologies and Systems (CTS).

[23] Mainetti L., Patrono L., Vergallo R. IDA-Pay: An Innovative Micro-Payment System Based on NFC Technology for Android Mobile Devices. Proceedings of the 20th International Conference on Software, Telecommunications and Computer Networks (SoftCOM).

[24] Urien P., Piramuthu S. Securing NFC Mobile Services with Cloud of Secure Elements (CoSE). Proceedings of the 5th International Conference on Mobile Computing, Applications and Services (MobiCASE).

[25] De Luna I.R., Ramos I., Montoro-Ríos F., Liébana-Cabanillas F.J. (2018). New perspectives on payment systems: Near field communication (NFC) payments through mobile phones. Mob. Commer. Concepts Methodol. Tools Appl..

[26] Yen-Jing N. (2019). Near field communication (NFC) mobile payment in Malaysia: A partial least square-structural equation modelling (PLS-SEM) approach. Int. J. Model. Oper. Manag..

[27] Park C.-H., Park C.-S. (2015). Public Key based Virtual Credit Card Number Payment System for Efficient Authentication in Card Present Transaction. J. Korea Inst. Inf. Secur. Cryptol..

[28] Al-Tamimi M., Al-Haj A. Online Security Protocol for NFC Mobile Payment Applications. Proceedings of the 2017 8th International Conference on Information Technology (ICIT).

[29] Madhoun N., Pujolle G. Security Enhancements in EMV Protocol for NFC Mobile Payment. Proceedings of the 2016 IEEE Trustcom/BigDataSE/ISPA.

[30] Haselsteiner E., Breitfuß K. Security in Near Field Communication (NFC). Proceedings of the RFIDSec’06 on RFID Security.

[31] Madlmayr G., Langer J., Kantner C., Scharinger J. NFC Devices: Security and Privacy. Proceedings of the 3rd International Conference on Availability, Reliability and Security (ARES), Technical University of Catalonia.

[32] Damme G.V., Wouters K., Preneel B. Practical Experiences with NFC Security on mobile Phones. Proceedings of the RFIDSec’09 on RFID Security.

[33] Nelson D., Qiao M., Carpenter A. (2013). Security of the Near Field Communication Protocol: An Overview. J. Comput. Sci. Coll..

[34] Levi M., Bissell P., Richardson T. (1991). The Prevention of Cheque and Credit Card Fraud.

[35] Bond M., Choudary O., Murdoch S.J. (2012). Chip and Skim: Cloning EMV Cards with the Pre-Play Attack. Comput. Res. Repos. (CoRR).

[36] Blaze M., Ioannidis J., Keromytis A.D. Offline Micropayments without Trusted Harware. Proceedings of the 5th International Conference on Financial Cryptography.

[37] Rivest R., Shamir A. PayWord and MicroMint: Two Simple Micropayment Schemes. Proceedings of the Security Protocols Workshop on Security Protocols.

[38] Lin I.C., Hwang M.S., Chang C.C. (2005). The General Pay-Word: A Micro-payment Scheme Based on n-dimension One-way Hash Chain. Des. Codes Cryptogr..

[39] Fan L.M., Liao J.X. (2007). Discrete Micropayment Protocol based on Master-Slave Payword Chain. J. China Univ. Posts Telecommun..

[40] Fan C.I., Liang Y.K., Wu C.N. An anonymous fair offline micropayment scheme. Proceedings of the 2011 International Conference on Information Society (i-Society).

[41] Yen S.M., Lin H.C., Chen Y.C., Hung J.J., Wu J.M. (2014). PayStar: A Denomination Flexible Micropayment Scheme. J. Inf. Sci..

[42] Kim S., Lee W. A PayWord-based Micropayment Protocol Supporting Multiple Payments. Proceedings of the 12th International Conference on Computer Communications and Networks (ICCCN).

[43] Esmaeeli A., Shajari M. MVPayword: Secure and Efficient Payword-based Micropayment Scheme. Proceedings of the 2nd International Conference on the Web Technologies (ICADIWT).

[44] Huszti A. Multi-Vendor PayWord with Payment Approval. Proceedings of the International Conference on Security and Management (SAM).

[45] Yang M.H. (2014). Security Enhanced EMV-based Mobile Payment Protocol. J. Sci. World J..

[46] Liu M., Xin Y., Yang Y., Niu X. Security Mechanism Research of EMV2000. Proceedings of the 2007 IEEE/WIC/ACM International Conferences on Web Intelligence and Intelligent Agent Technology–Workshops (WI–IATW).

[47] Murdoch S.J., Drimer S., Anderson R., Bond M. “Chip and PIN is Broken”. Proceedings of the 2010 IEEE Symposium on Security and Privacy (SP).

[48] Hancke G.P. Practical Eavesdropping and Skimming Attacks on High-frequency RFID Tokens. Proceedings of the 2010 Workshop on RFID Security.

[49] Bhole V.A., More R.R., Khadke N.C. (2007). Security in Near Field Communication (NFC) Strengths and Weaknesses. Proceedings of the 2nd National Conference on Emerging Trends in Information Technology (EIT).

[50] (2008). ITU-T Recommendation X.509 Information Technology—Open System Interconnection—The Directory Public-Key and Attribute Certificate Frameworks.

[51] Gong L., Needham R., Yahalom R. Reasoning about belief in cryptographic protocols. Proceedings of the IEEE Computer Society Symposium on Research in Security and Privacy.

